# Investigation of the functions of *n*-3 very-long-chain PUFAs in skin using *in vivo* Atlantic salmon and *in vitro* human and fish skin models

**DOI:** 10.1017/S0007114523001150

**Published:** 2023-12-14

**Authors:** Martina Torrissen, Elisabeth Ytteborg, Harald Svensen, Iren Stoknes, Astrid Nilsson, Tone-Kari Østbye, Gerd Marit Berge, Marta Bou, Bente Ruyter

**Affiliations:** 1 Nofima (Norwegian Institute of Food, Fisheries and Aquaculture Research), 1432 Ås, Norway; 2 Epax Norway, 6006 Ålesund, Norway; 3 NMBU (Norwegian University of Life Sciences), 1433 Ås, Norway

**Keywords:** Very-long-chain PUFA (VLC-PUFA), *n*-3, Fatty acids, Skin, Wound healing, Development, Atlantic salmon, Human skin model

## Abstract

The purpose of this study was to investigate the effect of dietary *n*-3 very-long-chain PUFA (*n*-3 VLC-PUFA) on the maturation and development of skin tissue in juvenile Atlantic salmon *(Salmo sala*r) *in vivo*, as well as their effects on skin keratocyte and human skin fibroblast cell migration *in vitro.* Atlantic salmon were fed different dietary levels of *n*-3 VLC-PUFA from an initial weight of 6 g to a final weight of 11 g. Changes in skin morphology were analysed at two time points during the experiment, and the effects on skin tissue fatty acid composition were determined. Additionally, *in vitro* experiments using human dermal fibroblasts and primary Atlantic salmon keratocytes were conducted to investigate the effect of VLC-PUFA on the migration capacity of the cells. The results demonstrated that increased dietary levels of *n*-3 VLC-PUFA led to an increased epidermis thickness and more rapid scale maturation in Atlantic salmon skin *in vivo*, leading to a more mature skin morphology, and possibly more robust skin, at an earlier life stage. Additionally, human skin fibroblasts and salmon skin keratocytes supplemented with *n*-3 VLC-PUFA *in vitro* showed more rapid migration, indicating potentially beneficial effects of VLC-PUFA in wound healing. In conclusion, VLC-PUFA may have beneficial effects on skin tissue development, function and integrity.

The skin is the largest organ in all vertebrates^(1)^ and serves as a protective barrier, separating the body’s internal milieu from the external environment. Disruption of this barrier can lead to infection, since open wounds can act as entry portals for pathogens; therefore, a strong barrier and rapid wound healing are highly important whenever this barrier is disrupted^(2)^.

Both mammalian and fish skin comprise three main structural compartments: epidermis, dermis and hypodermis^(3–6)^. The epidermis is the outermost layer of the skin, and its primary functions are to provide a physical barrier against pathogen invasion and act as a water-permeable barrier that regulates the amount of water released from the body through transepidermal water loss, which is essential for land-living animals to avoid death by dehydration^(7,8)^. The skin of aquatic vertebrates also serves as a particularly important first line of defence against the environment as the aquatic environment is rich in pathogens^(9)^. There are four major cell types in the epidermis, of which the most abundant types are keratinocytes in human skin and keratocytes in fish skin. Keratinocytes and keratocytes both form a tight barrier and play essential roles in defence. In fish, this barrier protects against the environment, entry of pathogens, and mechanical damage and serves an important role in osmoregulation (reviewed in Sveen *et al.*, 2020)^(3)^. In terrestrial mammals, this barrier also serves an important additional function in heat regulation and protecting against loss of water^(10)^. The dermis is the second layer and is composed of elastic and fibrous tissue, making it the most impenetrable layer of the skin^(11)^. The major cells in the dermis are fibroblasts^(12)^, and in addition to being responsible for tissue homoeostasis under normal physiological conditions, fibroblasts play a critical role in wound healing. Dermal fibroblasts located at the wound edges can become myofibroblastic and are key participants in tissue repair as they provide the contractile forces that bring the wound edges together^(13,14)^. Finally, the hypodermis predominantly comprises adipocytes (fat cells), and its main functions are fat storage and insulation^(4)^.

The structural and functional similarities between mammalian and fish skin make fish an interesting model species for studies of skin development in vertebrates. The skin of Atlantic salmon matures during the early parr life stage, making this life stage a particular good model for studies of skin development and function in vertebrates. The model allows repeated skin tissue biopsies which enables a comprehensive investigation of skin development within a very short period of time, studies which are very difficult or not possible to conduct in humans^(4)^. The most obvious difference between mammalian and teleost fish skin is that teleost fish skin has living cells covered with mucus in the epidermis layer of the skin, whereas human epidermis is covered in dead keratinised cells. In addition, the skin of many fish species has scales, whereas mammalian skin may have hair^(11,15)^. The mucus secreted by mucus cells covers the epidermis and serves to protect fish from infectious pathogens. The keratocytes covering the surface of fish skin will within hours after wounding cover the wounded area with a new protective layer of cells through rapid cell migration from the surrounding wound margins^(3)^. Fibroblasts also play an essential role in the regeneration of connective tissue in fish skin and comprise part of the connective tissue known as granulation tissue, which typically grows from wound borders to replace damaged tissue^(3)^. Understanding fish skin is important also from an aquaculture perspective, as compromised skin integrity of farmed Atlantic salmon has major economical and animal welfare implications^(16)^.

PUFA play major roles in skin barrier function and integrity. Burr and Burr^(17)^ were the first to reveal their essential role in rats fed a diet deprived of dietary fats, which resulted in skin symptoms, such as scaly skin and increased transepidermal water loss. Since then, several *in vivo* studies have demonstrated that consumption of PUFA improves skin barrier function in the presence of essential fatty acid deficiency or skin disease^(18,19)^. The anti-inflammatory properties of *n*-3 fatty acids from fish oils are well established and have been shown to influence the production of eicosanoids, which are considered key regulators of inflammation^(20)^. Studies have shown greater epithelialisation of blisters in groups receiving *n*-3 fatty acid supplementation compared with controls, suggesting improved wound healing, in addition to reduced wound infections in humans^(21)^. Additionally, acellular fish skin, which is a biomaterial used as a natural scaffold^(22)^, has gained increased attention in cellular and tissue-based therapies to treat chronic wounds, and its promising effects are most likely attributable to *n*-3 fatty acids^(23)^. *In vitro* keratinocyte wound models in which the constituent fatty acids from acellular fish skin are delivered to the wound have also demonstrated faster wound closure^(23)^. A possible hypothesis is that marine *n*-3 fatty acids increase the migration of cells to the wound bed by inducing specialised pro-resolving metabolic pathways, resulting in lower levels of inflammation in a similar manner to that of the downstream effects seen with non-steroidal anti-inflammatory drugs^(23)^. However, the exact mechanisms remain to be elucidated.

Several studies have indicated critical tissue-specific roles of very-long-chain fatty acids (VLC-FA), which are defined as fatty acids with a chain length of ≥ 24 carbon atoms, including in skin tissue^(24–27)^. This stems from the finding that mutations in the elongase of very long-chain fatty acids-4 (*ELOVL4*) gene, that encodes the ELOVL4 protein, which is responsible for mediating the biosynthesis of these fatty acids, is associated with several tissue-specific disorders^(28)^. VLC-FA are generally not obtained via the diet, and thus mutations in *ELOVL4* results in a lack of these fatty acids in tissues where they are normally present and consequently disorders of different magnitudes, indicating their importance. Studies in skin tissue revealed that loss of functional ELOVL4 protein is associated with skin disorders, defective skin permeability barrier, and has been shown to be lethal during early development^(24–27)^. The severity of these conditions indicates that VLC-FA play an essential role in animals. We have previously demonstrated deposition of *n*-3 VLC-PUFA in skin tissue after dietary supplementation in Atlantic salmon (*Salmo salar*) and mice^(29)^, which may indicate a role of VLC-PUFA in skin. This is an interesting finding, as the importance of VLC-FA in skin has mostly been examined in relation to very-long-chain SFA (VLC-SFA), while the role of VLC-PUFA remains unclear^(25,27,29)^. New studies of the specific function(s) of VLC-PUFA and their metabolic products are needed, particularly if dietary supplements with these compounds could play a role in the treatment of dermatological diseases and conditions.

The present study aimed to investigate if supplementing different levels of *n*-3 VLC-PUFA in the diet would affect skin tissue fatty acid composition and skin development of Atlantic salmon. Furthermore, cell culture experiments with primary keratinocytes from Atlantic salmon and human dermal fibroblasts were performed to elucidate the potential functions of VLC-PUFA *in vitro*, particularly on cell proliferation and migration, in these cell types.

## Materials and methods

### Production of *n*-3 very-long-chain-PUFA concentrates for Atlantic salmon feeding trial and cell culture experiments

Two *n*-3 VLC-PUFA concentrates, VLC-Conc1 and VLC-Conc2, were made from an anchovy fish oil distillation fraction by hydrolysis, precipitation with LiOH and distillation^(30)^ as previously described^(29)^. Both VLC-Conc1 and VLC-Conc2 were produced by Epax Norway AS. VLC-Conc1 was used for the salmon feeding trial, and VLC-Conc2 was used for the cell culture experiments. A synthetic VLC-PUFA 26:6 *n*-3, custom-made and purchased from BOC Sciences, was used in the cell culture experiments using primary keratocytes from Atlantic salmon. The fatty acid composition of the concentrates is presented in Table 1. The purpose of using different concentrates in the cell culture experiments and the salmon feeding trial was to single out the VLC-PUFA effect from effects of other fatty acids in the *in vitro* studies. Therefore, either a VLC-PUFA concentrate enriched with only unsaturated fatty acids with > 22 carbon atoms was used or a pure 26:6 *n*-3 substrate. The salmon feed, on the other hand, already consisted of many fatty acids other than the VLC-PUFA, and therefore there is no need to use a VLC-PUFA concentrate devoid of SFA and ≤ 22 carbon MUFA and PUFA.

**Table 1.** Fatty acid composition of VLC-PUFA concentrates (VLC-Conc1 and VLC-Conc2), presented as percentage (%) of total fatty acids*

	VLC-Conc1 in salmon feed (%)	VLC-Conc2 in cell culture trials (%)
14:0	0·20	nd
16:0	3·33	nd
18:0	1·03	nd
24:0	nd	nd
26:0	nd	nd
28:0	0·12	nd
Sum of SFA	4·68	nd
16:1	1·59	nd
18:1 *n*-9 + *n*-7	7·28	nd
20:1	0·56	nd
22:1	2·12	nd
24:1	2·78	1·29
26:1	0·23	2·97
28:1	0·06	nd
Sum of MUFA	14·62	4·26
18:2 *n*-6	0·69	nd
20:4 *n*-6	0·84	nd
22:5 *n*-6	nd	nd
Sum of *n*-6 PUFA	1·53	nd
18:3 *n*-3	0·40	nd
18:4 *n*-3	1·31	nd
20:4 *n*-3	0·58	nd
20:5 *n*-3	12·88	nd
21:5 *n*-3	0·37	nd
22:5 *n*-3	6·72	0·20
22:6 *n*-3	24·68	0·68
Sum of *n*-3 LC-PUFA	46·94	0·88
24:4 *n*-3	0·68	0·33
24:5 *n*-3	2·91	1·47
24:6 *n*-3	1·51	0·47
26:4 *n*-3	0·77	nd
26:5 *n*-3	1·86	4·34
26:6 *n*-3	2·47	9·56
26:7 *n*-3	0·40	0·95
28:4 *n*-3	0·33	nd
28:5 *n*-3	0·50	2·04
28:6 *n*-3	0·79	5·31
28:7 *n*-3	0·15	1·42
28:8 *n*-3	9·35	62·25
Sum of C30 fatty acids	0·06	5·03
Sum of *n*-3 VLC-PUFA	21·78	93·17
Sum of VLC-FA	24·97	97·43
Others†	0·68	nd
Total	90·23	98·31

ND, not detected; VLC-PUFA, very-long-chain PUFA.

* Analytical methods used detects fatty acids ≤ C30.

† Others include 16:4, 24:2, 26:3 and 28:2.

### Dietary supplementation of Atlantic salmon with increasing levels of *n*-3 very-long-chain-PUFA

A 4-week *in vivo* feeding trial using Atlantic salmon (Salmo Breed) was conducted in freshwater tanks at Nofima’s Aquaculture Research facilities at Sunndalsøra, Norway. Five different experimental diets that were produced at the Nofima Feed Technology centre in Bergen, Norway, were tested using three tanks per diet group and 100 fish per tank. During the study period, the fish grew from an initial weight of approximately 6 g to a final weight of approximately 11 g. The basal diet consisted of 50 % fishmeal and 10 % each of wheat, wheat gluten, and soya protein concentrate, in addition to minerals and vitamin mixture (Table 2). The diets were isoenergetic and contained 50 % protein and 20 % lipid. The five different experimental diets were supplemented with increasing levels (0–10 %) of VLC-Conc1, while levels of EPA and DHA were kept constant. The amount of fish oil was reduced from 13 % to 0 % as the amount of VLC-Conc1 and rapeseed oil increased in the diets. The composition of the basal diet was identical for all groups, except for the fatty acid composition of the oil coating of the basal diet, which was coated on the pellets with a vacuum coater in the final production step. The ingredient and fatty acid compositions of the diets are shown in Tables 2 and 3, respectively. We aimed at optimal voluntary feed intake in each tank. By controlled over-feeding, we secured that all fish had access to sufficient amount of feed throughout the experiment.

**Table 2.** Ingredient composition of the diets, presented as g/100 g feed. Diet groups are named according to the percentage VLC-Conc1 included in the feed

	VLC-Conc1 (%)				
	0	2·5	5·0	7·5	10
Fishmeal (Micromesistius poutassou)*	50·00	50·00	50·00	50·00	50·00
Wheat†	10·00	10·00	10·00	10·00	10·00
Wheat gluten‡	10·00	10·00	10·00	10·00	10·00
Soya protein concentrate§	10·00	10·00	10·00	10·00	10·00
Choline chloride||	0·50	0·50	0·50	0·50	0·50
Vitamin mix¶	2·00	2·00	2·00	2·00	2·00
Monosodium phosphate**	2·00	2·00	2·00	2·00	2·00
Carophyll pink††	0·01	0·01	0·01	0·01	0·01
Mineralmix‡‡	0·50	0·50	0·50	0·50	0·50
Fish oil (Clupea harengus)*	13·54	10·15	6·76	3·38	0·00
Rapeseed oil§§	0·00	1·25	2·50	3·75	4·99
DHA (EPAX 0460 TGN)||||	1·36	1·02	0·68	0·34	0·00
VLC-Conc1¶¶	0·00	2·50	5·00	7·50	10·00

VLC-PUFA, very-long-chain PUFA.

* Vedde AS (Norway).

† Norgesmøllene AS (Norway).

‡ Tereos Syral (France).

§ Agrokorn (Germany).

|| Vilomix (Norway).

¶ Normin (Norway). Provided per 100 grams of feed: vitamin D, 300 mg; vitamin E, 16 mg; thiamin, 2 mg; riboflavin, 3 mg; pyridoxine-HCl, 3 mg; vitamin C, 20 mg; calcium d-pantothenate, 6 mg; biotin, 0·11 mg; folic acid, 1 mg; niacin, 20·1 mg; cobalamin, 0·005 mg; vitamin K3, 2 mg.

** Normin (Norway).

†† DSM (The Netherlands).

‡‡ Normin (Norway), Provided per 100 g of feed: K, 80 mg; Mg, 75 mg; Zn, 12 mg; Fe, 6 mg; Mn, 3 mg; Cu, 0·6 mg; Se, 0·03 mg.

§§ Emmelev (Denmark).

|||| Epax Norway AS (Norway).

¶¶ Epax Norway AS (Norway). 0·00, 2·50, 5·00, 7·50 and 10·00 of concentrate corresponds to 0·00, 0·54, 1·09, 1·63 and 2·18 VLC-PUFA in g/100 g feed, respectively.

**Table 3.** Fatty acid composition of the diets, presented as grams of fatty acid/100 g feed. Diet groups are named according to the percentage VLC-Conc1 included in the feed

	VLC-Conc1 (%)				
	0	2·5	5	7·5	10
14:0	0·98	0·76	0·54	0·34	0·14
16:0	2·44	2·10	1·74	1·42	1·10
18:0	0·32	0·30	0·28	0·26	0·24
20:0	0·02	0·04	0·02	0·04	0·04
22:0	0·02	0·02	0·02	0·02	0·02
Sum of SFA	3·76	3·22	2·60	2·06	1·54
16:1 *n*-7	0·98	0·82	0·62	0·44	0·28
18:1 *n*-9 + *n*-7 + *n*-5	2·74	3·18	3·46	3·94	4·16
20:1 *n*-9 + *n*-7	2·16	1·74	1·26	0·84	0·44
22:1 *n*-11 + *n*-9 + *n*-7	2·48	2·00	1·50	1·00	0·54
24:1 *n*-9	0·14	0·16	0·20	0·22	0·24
Sum of MUFA	8·50	7·90	7·04	6·46	5·66
18:2 *n*-6	0·66	0·84	1·4	1·24	1·40
18:3 *n*-6	0·02	0·02	0·02	0·02	0·02
20:2 *n*-6	0·04	0·04	0·04	0·02	0·02
20:3 *n*-6	0·02	0·02	0·02	0·02	0·02
20:4 *n*-6	0·08	0·08	0·08	0·08	0·08
22:4 *n*-6	0·02	0·02	0·02	0·04	0·02
Sum of *n*-6 PUFA	0·84	1·02	1·20	1·42	1·56
18:3 *n*-3	0·18	0·30	0·38	0·50	0·58
18:4 *n*-3	0·38	0·32	0·26	0·22	0·16
20:3 *n*-3	0·02	0·02	0·02	0·02	0·02
20:4 *n*-3	0·12	0·10	0·08	0·10	0·06
20:5 *n*-3 (EPA)	1·30	1·32	1·28	1·30	1·26
21:5 *n*-3	0·08	0·08	0·08	0·08	0·06
22:5 *n*-3	0·30	0·36	0·44	0·52	0·58
22:6 *n*-3 (DHA)	2·36	2·38	2·38	2·40	2·38
Sum of *n*-3 PUFA	4·74	4·88	4·92	5·10	5·10
Sum of all PUFA	5·68	6·00	6·22	6·58	6·72
Sum EPA + DHA	3·66	3·70	3·66	3·70	3·64
24:4 *n*-3*	0·00	0·02	0·03	0·5	0·07
24:5 *n*-3*	0·00	0·07	0·15	0·22	0·29
24:6 *n*-3*	0·00	0·04	0·08	0·12	0·16
26:4 *n*-3*	0·00	0·02	0·04	0·06	0·08
26:5 *n*-3*	0·00	0·05	0·09	0·14	0·19
26:6 *n*-3*	0·00	0·06	0·12	0·19	0·25
26:7 *n*-3*	0·00	0·01	0·02	0·03	0·04
28:4 *n*-3*	0·00	0·01	0·02	0·02	0·03
28:5 *n*-3*	0·00	0·01	0·03	0·04	0·05
28:6 *n*-3*	0·00	0·02	0·04	0·06	0·08
28:7 *n*-3*	0·00	0·00	0·01	0·01	0·02
28:8 *n*-3*	0·00	0·23	0·47	0·70	0·94
Sum of VLC-PUFA*	0·00	0·54	1·09	1·63	2·18

VLC-PUFA, very-long-chain PUFA.

* Values calculated based on inclusion levels of VLC-Conc1 in the feed.

Growth rates were calculated based on the average weights of the fish in each tank as follows, where W_0_ represents the starting weight (g), W_1_ represents the final weight (g), t represents the number of days, and d° represents the degree-days (sum of average temperature for all feeding days):

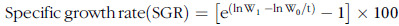




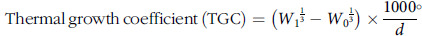




### Sampling and sample preparation

Ten fish were randomly selected from each tank and euthanised by overdose (0·05–0·08 g/l) of anaesthetic (Metacain, MS-222) at three different time points: start of the trial (0 d); midway (18 d); and end of the trial (28 d). Standardised tissue samples of skin from the area above the lateral line and underneath the dorsal fin were taken from ten fish and fixed in neutral buffered 10 % formalin solution (Sigma-Aldrich). Skin samples were also excised and frozen in liquid N_2_ before storing at –80°C until used later to analyse lipid composition. At the final sampling, all the fish in each tank were bulk-weighed.

### Histology

Skin samples taken at the start, midway and final sampling (days 0, 18 and 28, respectively) were dissected and placed in tissue-embedding cassettes (Simport). Tissue processing was performed using an automated tissue processor (TP1020, Leica Biosystems, Nussloch GmbH) in which the samples were dehydrated using 100 % alcohol and a clearent xylene bath, before infiltration in melted paraffin at 60°C (Merck KGaA). Paraffin-embedded tissue samples were cut into 5-µm sections using a Microtome (Leica RM 2165), mounted on polysin-coated slides (VWR, Avantor) and dried overnight at 37°C. The sections were deparaffinised and rehydrated, and staining was performed using an automated special stainer (Autostainer XL Leica Biosystems, Nussloch GmbH Paraffin sections were stained with haematoxylin–eosin (H&E) and Von Kossa (Sigma Aldrich). All slides were examined using a light microscope slide scanner (Leica Microsystems) and manually evaluated using an Aperio Image Scope (Leica).

### *In vitro* cell culture of primary Atlantic keratocytes and human dermal fibroblasts

#### Fatty acid substrates for cell culture experiments

In the cell culture experiments, fatty acids were added to the growth media in the form of their Na salts bound to bovine serum albumin (2·7:1 molar ratio) as previously described by Bou *et al.* ^(31)^. The pH was adjusted to 7 using NH_4_OH. DHA was purchased from Sigma (Sigma Aldrich). The *n*-3 VLC-PUFA 26:6 *n*-3 and VLC-Conc2 were both prepared as 1-mM stock solutions, and DHA was prepared as an 8-mM stock solution. All fatty acid solutions were stored at –80°C.

#### Atlantic salmon keratocytes: cells and media

Growth medium was prepared as L-15 (11544436, Fischer Scientific) supplemented with 5 % fetal bovine serum (F2442, Sigma-Aldrich), 1 % anti/anti (A5955, Sigma-Aldrich; 15240062 Fischer Scientific) and 1 % HEPES (H0887, Sigma-Aldrich). The test substrates added were either 25 ng/ml fibroblast growth factor (FGF) (106 096–93–9, Sigma-Aldrich), 10 µM VLC-PUFA (26:6 *n*-3) or 20 µM VLC-PUFA (26:6 *n*-3). Controls were left untreated. FGF is known to induce cell proliferation and may be considered a positive control. The cells were incubated at 12°C without CO_2_.

#### Cell migration of primary Atlantic salmon keratocytes from scales

An *in vitro* study using primary cell cultures of salmon scales was performed as previously described^(32)^ to examine whether the *n*-3 VLC-PUFA 26:6 *n*-3 affected salmon keratocyte migration. In brief, seven freshwater Atlantic salmon were transported from NIVA (Aas) to Nofimas’ research facilities (Aas) in an enclosed plastic bag filled with oxygen and moderately anesthetised with Aqui-S (Scanvacc, Aqui-S vet.). The fish were exposed to an overdose of anaesthetic (Aqui-S) according to the manufacturers’ protocol, for approximately 5 min before being sacrificed by a blow to the head. Forceps were used to pick single scales from the dorsal part of the lateral line of the fish and placed in 12-well tissue culture plates (Thermo-Fisher Scientific). Each well contained 2 ml of growth medium with or without test substrates, which were added at the time of isolation.

The day after isolation, all wells were inspected under a microscope (Leica Microsystems, Nussloch GmbH) to determine the migration potential of the scales as previously described^(32)^. In brief, scales with and without cells migrating from the scales were evaluated and counted. As attachment is a prerequisite for cell migration, only scales that were attached to the cell plates were included and the percentage of scales with cell migration was calculated. Images were taken using a Canon camera EOS 550D (Canon Inc.). All scales with migration were counted to determine the immediate effect of the VLC-PUFA on cell migration. Inspection, counting and photographing were repeated at three different time points: 24 h, 30 h and 57 h after plucking the scales. The mean percentage of scales with cell migration from each fish was used in the calculations, where the individual fish was used as the experimental unit.

#### Human dermal fibroblasts: cells and media

The ATCC PCS-201–012 commercial human dermal fibroblast cell line was cultured in low-glucose Dulbecco’s modified Eagle’s medium (Thermo-Fisher Scientific) supplemented with 10 % fetal bovine serum (F7525, Sigma-Aldrich) and 0·1 % anti/anti (A5955, Sigma-Aldrich; 15240062 Fischer Scientific) in tissue culture flasks (Thermo-Fisher Scientific). The cells were maintained at 37°C in a humidified atmosphere with 5 % CO_2_ and subcultivated when they reached confluence. Cells were used for experiments between passages 4 and 6 in this study, and 3 µM VLC-Conc2 or 3 µM DHA were added as test substrates in the experiments. The control group was treated with albumin in phosphate buffer at a level comparable to that used in the VLC-Conc2 group.

#### *In vitro* culture of human dermal fibroblasts for fatty acid composition analysis

For fatty acid composition analysis, cells were seeded in T25 flasks (Thermo-Fisher Scientific) at a density of 125·000 cells/flask in 5 ml of culture media and incubated until approximately 70 % confluence (Leica DM IL LED light microscope, Leica Microsystems, Nussloch GmbH). Cells were then washed once in 5 ml of PBS, and test substrates of either 3 µM VLC-Conc2 or 3 µM DHA were added prior to further incubation. The control group was treated with albumin in phosphate buffer at levels comparable to those of the VLC-Conc2 and DHA groups. Three replicates per substrate group were included. The following day, cells were washed once in 5 ml of PBS, fresh media containing the test substrates were added and the flasks were further incubated overnight. The flasks were then washed twice in PBS with 1 % bovine serum albumin and then washed twice in PBS. Cells were loosened in 1 ml of PBS using a rubber scraper before being transferred to Eppendorf tubes and centrifuged at 550 × *g* for 5 min. After centrifugation, as much PBS as possible was removed without touching the cell pellet, which was then stored at –80°C for later analysis of fatty acid composition.

#### Scratch assay of human dermal fibroblasts, *in vitro* cell migration and wound healing model

A scratch assay was performed to study whether the *n*-3 VLC-PUFA concentrate (VLC-Conc2) affected cell migration. Cells were seeded at 1 × 10^4^ cells/cm^2^ in 8·87 cm^2^ wells (TC Plate 6 Well, Standard, F, Sarstedt) in 3 ml of growth media. The cell migration experiment was repeated three times (*n* 3), with 6–9 parallels per replicate (giving a total of 18 for the VLC-Conc2 and DHA groups, and 21 for the control group). Test substrates were added when the cells reached approximately 70 % confluency, and the plates were further incubated overnight. When the cells reached approximately 90 %–100 % confluency, a scratch was created by pulling a pipette tip over the centre of the cell monolayer (Biosphere® Filter Tips, 70·750·211, 2–200 µl), tracing a ruler to ensure a straight line. Culture media containing the substrates were replaced with new media after washing once with 2 ml of PBS to remove loose cells. The wells were then photographed immediately after the scratch was created and then at several time points (2–4-h intervals) up to 14 h post-scratching (HPS). Migration of cells into the scratched area was examined and photographed using the Personal Automated Lab Assistant (Paula, Leica Microsystems). Cell migration was quantified as the size of the scratched area relative to the initial scratch size using the open-source image analysis software package Fiji/ImageJ (Fiji for Mac OS X).

### Fatty acid composition analysis

For the fatty acid composition analysis, total lipids were extracted from homogenised tissue or sonicated extracts of cells following the method previously described by Folch *et al.* ^(33)^. For skin tissue, three pooled samples of ten individual tissue samples (ten fish from each of the triplicate tanks per diet group, thirty fish in total) were analysed for lipid composition per diet group. For cells, three individual cell culture flasks per treatment group were analysed.

To determine the lipid class composition of the skin tissue, the Folch chloroform lipid extract was evaporated under N_2_ gas, and the residual lipid extract was redissolved in hexane (VWR). Phospholipids (PL) were separated by TLC using the method described by Bou et al^(34)^. In brief, TLC plates (Watman K6 – Silica Gel 60 Å, 0·25-mm film thickness, 20 × 20 cm; VWR) were preconditioned in methanol in a separation chamber under a fume hood and dried at 120°C for 20 min. Lipid fractions were then applied to the plates for migration and placed in a TLC separation chamber. PL were separated using a mixture of petroleum ether, diethyl ether and acetic acid (113:20:2, *v*/*v*/*v*) as the mobile phase. The plates were then dried in a fume hood, sprayed with 2 % 2–7-dichlorofluorescein in ethanol and dried again. UV light (366 nm) was used to detect the lipid classes, which appeared as yellow spots when placed under UV light. Spots were marked while under the UV light, and the plates were then brought back to the fume hood and the lipid classes were identified by comparing with known standards (Sigma Chemical Co.). The marked areas corresponding to the PL fraction was scraped and transferred to glass tubes and dissolved in Arvidson’s solution. The tubes were then capped and mixed using a vortex mixer, frozen overnight at −40°C and then trans-methylated.

The fatty acid composition of the separated lipid group was analysed using the method described by Mason and Waller^(35)^. Extracts were trans-methylated overnight using 2′,2′-dimethoxypropane, methanolic HCl and benzene at room temperature. For cells, fatty acid methyl esters were prepared by adding hexane + 0·1 % BHT (1 ml) to the reaction tubes followed by a solution of saturated NaHCO_3_ (2 ml). The organic layer was transferred to glass tubes and evaporated at 60°C under N_2_ gas. The residues were redissolved in hexane + 0·1 % BHT (50 µl) and transferred to GC vials.

The total fatty acid composition of the PL fraction from the skin tissue and total fatty acids from the cells were determined as follows. Methyl esters were separated and analysed in a GC (Hewlett Packard 6890; HP) with a split injector using an SGE BPX70 capillary column (length, 60 m; internal diameter, 0·25 mm; and film thickness, 0·25 μm; SGE Analytical Science), flame ionisation detector and HP Chem Station software. He was used as the carrier gas, and both the injector and detector temperatures were 280°C. The oven temperature started at 50°C for 1·2 min and was then increased to 170°C at a rate of 4°C/min, then to 200°C at a rate of 0·5°C/min, and finally to 280°C at a rate of 10°C/min. The individual fatty acid peaks were identified by comparing the retention times with validated standards; GLC reference 463 and 85 (Nu-chek Prep). The absolute amount of fatty acid per gram of tissue or cells was calculated using 23:0 methyl ester as an internal standard (IS). Unidentified peaks were not included in the total fatty acid calculation.

The VLC-PUFA composition of the cells was analysed using a Scion 436-GC with a split/splitless injector (splitless 1 min) with a Restek Rxi-5ms capillary column (length, 30 m; internal diameter, 0·25 mm; and film thickness, 0·25 mM), flame ionisation detector and CompasCDS Software. Hydrogen was used as the carrier gas, with split injection and detector temperatures of 250°C and 270°C, respectively. The oven temperature started at 90°C for 1·0 min and was ramped up to 200°C at a rate of 45°C/min, then to 280°C at a rate of 2·5°C/min, and finally to 340°C at a rate of 10°C/min. Data were calculated by comparing the area peaks to the known amount of 23:0 IS added to the samples and presented as area percentage of the selected fatty acids.

### Gene expression analysis

Fibroblasts were harvested for gene expression analysis immediately after creating the scratch (0 HPS) in the scratch assay (*n* 6 for all groups), and 1 d post-scratching (1 DPS; *n* 6 for the VLC-Conc2 and DHA groups, *n* 9 for the control group). In brief, wells were washed once in PBS before being lysed using RNeasy lysis buffer. A sterile cell scraper with a two-position blade (Starstedt) was used to loosen the cells, which were then transferred to QIAshredder spin columns, centrifuged at 550 × *g* for 5 min and stored at –80°C for later analysis.

Total RNA was isolated from fibroblasts using RNeasy Plus Mini Kit (Qiagen) according to the manufacturer’s protocol. All samples were treated with DNase I (Invitrogen) to remove genomic DNA. The concentration and purity of RNA were evaluated using a NanoDrop 1000 Spectrophotometer (NanoDrop Technologies). cDNA was synthesised from 725 ng of RNA in a 20-μl reaction volume using Taqman RT reagents (Applied Biosystems) under the following conditions: 25°C for 10 min, 37°C for 30 min to synthesise cDNA and 95°C for 5 min to terminate the cDNA synthesis reaction. The quantitative PCR reaction mixture consisted of 4 μl of diluted (1:10) cDNA, 1 μl of forward and reverse primers (final concentration of 0·5 μM) and 5 μl of PowerUp SYBR Green Master Mix (Applied Biosystems). A standard curve was included for each primer pair to evaluate the primer efficiency. All the primers used are listed in Table 4. All samples were analysed in parallel, and non-template and non-enzyme controls were included. The quantitative PCR reaction was run on a QuantStudio 5 instrument (Thermo-Fisher Scientific) under the following conditions: initial denaturation, 95°C for 20 s; amplification, 40 cycles at 95°C for 1 s and 60°C for 20 s; melting, 95°C for 1 s and 60°C for 20 s; dissociation, 95°C for 1 s. *EF1A*, *RPOL2* and *GAPDH* were evaluated as reference genes using RefFinder^(36)^ to identify which was most stable. The relative gene expression level was calculated according to the DDCt method with efficiency correction (Pfaffl 2004) using *RPOL2* as a reference gene^(37)^.

**Table 4.** Primers used for quantitative PCR analysis of human dermal fibroblast genes

Gene	Accession no.	Forward primer (5'-3')	Reverse primer (5'-3')
*ELOVL4*	NM_022726·4	GGTCCATCGCAGATAAGCGT	ATTTTGGACCCAGCCACACA
*CERS2*	NM_181746·3	CTCTTGATGCCCTCCCCTTTG	GTGTAGCCACGTACAGCTCA
*IL8*	NM_000584	CAGAGACAGCAGAGCACACA	GGCAAAACTGCACCTTCACA
*FGF2*	NM_001361665·2	GCGACCCTCACATCAAGCTA	AGCCAGGTAACGGTTAGCAC
*VEGFa1*	NM_001025366·3	GGCAAAAACGAAAGCGCAAG	GAGGCTCCAGGGCATTAGAC
*TGFa*	NM_003236·4	TGTCTGCCATTCTGGGTACG	GCAGCAGTGTATCAGCACAC
*CPT1A*	NM_001876·4	GCAGCGTTCTTTGTGACGTT	AGGAGTGTTCAGCGTTGAGG
*EF1A*	NM_001402·5	GACACGTAGATTCGGGCAAGTCCA	CCATCTCAGCAGCCTCCTTCTCAA
*RPOL2*	NM_000937·4	GCGCAATGAGCAGAACGGCG	ACTTCTGCATGGCACGGGGC
*GAPDH*	NM_002046·3	ATCCCATCACCATCTTCCAGGAGC	AAATGAGCCCCAGCCTTCTCCAT

### Statistical analysis

Data are expressed as mean ± standard error of the mean (sem) or pooled sem. For the salmon feeding trial, weights and growth data were collected from three replicate tanks with 100 individual fish in each. Growth rates were calculated based on average fish weight in each tank. Tank values were used as experimental units (*n* 3). Ten fish per tank, giving a total of thirty fish per dietary group, were collected for fatty acid composition analysis. Tank values were used as experimental units (*n* 3), and linear regression models were used to evaluate the relationship between fatty acid tissue content and *n*-3 VLC-PUFA levels in the feed. For histology, five fish from each tank, giving a total of fifteen fish per treatment, were analysed. For the salmon keratocyte migration trial, data were collected from a total of six individual fish in the control group, seven in the FGF group, and five for each of the 10 uM VLC-PUFA and 20 uM VLC-PUFA groups. The individual fish were used as experimental units. For each fish, the number of scales with cell migration was divided by the number of attached scales (varying from 3–10 scales), and the mean of all wells for each fish was used in the calculations. For the human dermal fibroblast scratch assay trial, three separate trials were used, with separate thawing of the cell batch for each trial, where each test group includes 6–9 individual wells. Data are presented as mean ± sem of each trial, where *n* 3. Data for the human dermal fibroblasts gene expression analysis were collected from two separate scratch assay trials: one for the 0 HPS (*n* 6 in all groups) and another for the 1 DPS (*n* 6 in DHA and VLC-Conc2 groups, and *n* 9 in control group), where the experimental unit represents individual wells. One-way ANOVA was used to assess differences between the groups, and significant (*P* < 0·05) differences were ranked according to Tukey’s honest significant difference test. JMP Pro 13.1.0 (SAS Institute Inc., 1989–2019) and Microsoft Office Excel software were used for the statistical analyses, and GraphPad Prism 9.3.0 was used to create the figures.

## Results

### Atlantic salmon fed increasing dietary levels of *n*-3 very-long-chain-PUFA

#### Growth performance

The mean weights of the fish at the beginning and end of the trial are presented in Table 5, along with the SGR and TGC. The weight of the fish increased almost twofold during the trial, from a start weight of approximately 6 g to a termination weight of about 11 g. There was no significant difference in the final weights of the fish between the diet groups (*P* = 0·16); however, there was a moderately lower SGR of 1·87 in the 10 % VLC-Conc1 diet group compared with SGR of 2·17 in the control group with 0 % VLC-PUFA (*P* = 0·04), and an almost significant difference in TGC between the diet groups (*P* = 0·06).

**Table 5.** Mean weights in grams and growth rates of salmon fed the experimental diets, presented as mean values and pooled standard error of the mean (sem) (*n* 3 tanks, 100 fish per tank per diet group). Diet groups are named according to the percentage VLC-Conc1 included in the feed

	VLC-Conc1 (%)						
	0	2·5	5	7·5	10	Pooled sem	*P*
BW start	6·00	6·00	6·10	6·1	6·10	0·07	0·86
BW termination	11·00	10·60	10·70	11·10	10·20	0·24	0·16
SGR	2·17^a^	2·03^ab^	2·04^ab^	2·15^a^	1·87^b^	0·06	0·04*
TGC	1·20	1·13	1·13	1·20	1·03	0·04	0·06

BW, body weight; SGR, specific growth rate; TGC, thermal growth coefficient.

^a-b^Groups not sharing the same letter are significantly different.

* Significant difference (*P* < 0·05).

#### Fatty acid composition of skin phospholipids

The total fatty acid compositions of the skin PL and TAG fractions from salmon fed different dietary levels of VLC-Conc1 are presented in Tables 6 and 7, respectively. The skin PL fatty acid composition greatly reflected that of the feed. EPA and DHA levels were balanced between the diet groups, and the data showed no significant differences in the EPA (20:5 *n*-3) or DHA (22:6 *n*-3) content of the skin between the diet groups. As the inclusion level of rapeseed oil increased in the feed, the typical fatty acids found in this oil (18:1 *n*-9, 18:2 *n*-6 and 18:3 *n*-3) significantly increased in the PL fraction of the skin. It is worth noting that 18:1 *n*-9 was also present in VLC-Conc1. There were few significant differences in the TAG fraction of skin (Table 7). Similar to the PL fraction, data showed no significant differences in the EPA (20:5 *n*-3) or DHA (22:6 *n*-3) content in TAG between the diet groups. As the inclusion level of rapeseed oil increased in the feed, 18:3 *n*-3 increased in the TAG fraction of the skin. The typical North Atlantic fish oil fatty acid 22:1 *n*-11 significantly decreased as the dietary level of fish oil was reduced, thus also reflecting the fatty acid composition of the feed.

**Table 6.** Fatty acid composition of the PL fraction from salmon skin measured as mg fatty acid/g tissue and presented as mean values and pooled standard error of the mean (sem) (three tanks per diet group, with pooled samples of ten fish from each of the triplicate tanks per diet group, giving thirty fish in total from each diet group)†. Diet groups are named according to the percentage VLC-Conc1 included in the feed

	VLC-Conc1 (%)						
	0	2·5	5	7·5	10	Pooled sem	*P*
14:0	0·20	0·19	0·22	0·18	0·14	0·02	0·06
15:0	0·03	0·02	0·04	0·03	0·03	0·01	0·82
16:0	1·78	1·58	1·81	1·71	1·66	0·08	0·31
18:0	0·35	0·33	0·39	0·37	0·38	0·02	0·10
20:0	0·03	0·02	0·02	0·02	0·02	0·00	0·71
24:0	0·05	0·05	0·05	0·04	0·05	0·00	0·46
Sum of SFA	2·45	2·20	2·55	2·37	2·31	0·10	0·24
16:1 *n*-7	0·16	0·13	0·16	0·13	0·13	0·01	0·28
18:1 *n*-7	0·20	0·17	0·20	0·19	0·21	0·02	0·46
18:1 *n*-9	0·83	0·82	1·08	1·11	1·27	0·06	0·00*
20:1 *n*-9	0·19	0·16	0·17	0·15	0·13	0·01	0·04*
22:1 *n*-7	0·04	0·03	0·03	0·03	0·03	0·00	0·37
22:1 *n*-11	0·09	0·07	0·09	0·07	0·07	0·01	0·46
Sum of MUFA	1·50	1·38	1·74	1·68	1·84	0·11	0·07
18:2 *n*-6	0·15	0·16	0·21	0·24	0·29	0·01	< 0·01*
20:3 *n*-6	0·02	0·02	0·02	0·01	0·01	0·00	< 0·05*
Sum of *n*-6 PUFA	0·21	0·22	0·26	0·30	0·36	0·01	< 0·01*
18:3 *n*-3	0·03	0·04	0·06	0·07	0·09	0·00	< 0·01*
20:3 *n*-3	0·13	0·12	0·12	0·12	0·13	0·00	0·47
20:4 *n*-3	0·03	0·02	0·03	0·02	0·02	0·00	0·26
20:5 *n*-3	0·48	0·43	0·42	0·44	0·43	0·02	0·14
22:5 *n*-3	0·14	0·13	0·13	0·15	0·15	0·01	0·09
22:6 *n*-3	2·54	2·37	2·28	2·37	2·25	0·10	0·35
Sum of *n*-3 PUFA	3·35	3·11	3·04	3·18	3·07	0·13	0·50
Sum of EPA + DHA	3·02	2·80	2·70	2·81	2·68	0·12	0·32

PL, phospholipid; VLC-PUFA, very-long-chain PUFA.

* Significant difference (*P* < 0·05).

**Fig. 1.**
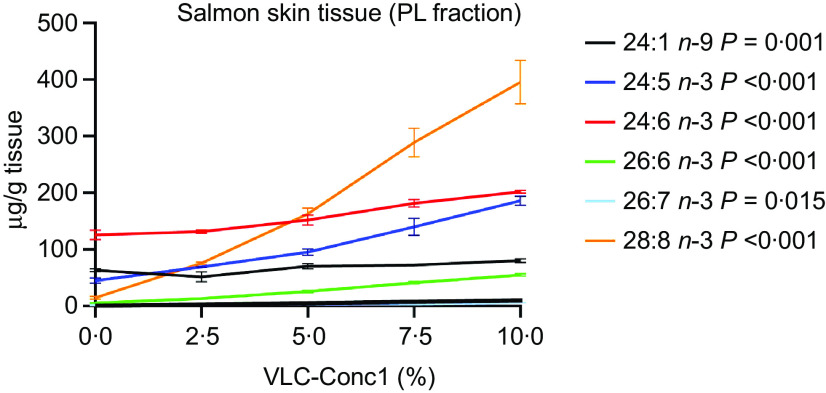
VLC-PUFA composition (µg/g tissue) in the PL fraction from the skin of salmon fed different levels (%) of VLC-PUFA (VLC-Conc1) in the diet. Data are presented as the mean values of three groups per diet group, consisting of ten individual skin samples in each group (*n* 3) and their standard error of the mean (sem). PL, phospholipid; VLC-PUFA, very-long-chain PUFA.

† VLC-PUFA composition is presented separately in Fig. 1 and Table 8.

**Table 7.** Fatty acid composition of the TAG fraction from salmon skin measured as mg/g tissue and presented as mean values and pooled standard error of the mean (sem) (three tanks per diet group, with pooled samples of ten fish from each of the triplicate tanks per diet group, giving thirty fish in total from each diet group)†. Diet groups are named according to the percentage VLC-Conc1 included in the feed

	VLC-Conc1 (%)						
	0	2·5	5	7·5	10	Pooled sem	*P*
14:0	4·05	3·39	3·49	3·12	3·14	0·31	0·27
15:0	0·30	0·25	0·26	0·23	0·23	0·02	0·26
16:0	11·88	10·45	10·94	10·27	10·37	0·88	0·69
17:0	0·22	0·19	0·21	0·21	0·19	0·02	0·81
18:0	2·45	2·24	2·40	2·31	2·39	0·19	0·94
20:0	0·17	0·19	0·18	0·17	0·19	0·02	0·86
24:0	0·19	0·21	0·20	0·21	0·22	0·01	0·86
Sum of SFA	19·26	16·79	17·68	16·52	16·64	1·46	0·66
14:1 *n*-5	0·18	0·15	0·14	0·13	0·13	0·02	0·33
16:1 *n*-5	0·16	0·16	0·15	0·16	0·13	0·04	0·98
16:1 *n*-7	4·46	3·81	3·99	3·75	3·54	0·34	0·42
16:1 *n*-9	0·21	0·17	0·16	0·20	0·18	0·02	0·38
17:1 *n*-7	0·29	0·19	0·19	0·17	0·17	0·04	0·22
18:1 *n*-7	2·63	2·40	2·55	2·46	2·50	0·20	0·93
18:1 *n*-9	14·13	13·14	15·34	15·49	16·74	1·18	0·31
18:1 *n*-11	0·41	0·53	0·26	0·41	0·40	0·08	0·27
20:1 *n*-7	6·04	4·78	4·61	3·94	3·71	0·38	0·01*
20:1 *n*-9	1·07	0·93	0·92	0·83	0·85	0·07	0·19
20:1 *n*-11	1·16	0·96	1·00	0·93	0·96	0·08	0·37
22:1 *n*-7	0·64	0·61	0·63	0·61	0·60	0·05	0·98
22:1 *n*-9	0·62	0·52	0·51	0·45	0·43	0·04	0·06
22:1 *n*-11	6·60	5·41	5·25	4·53	4·31	0·45	0·03*
24:1 *n*-9	0·56	0·56	0·68	0·71	0·73	0·04	0·05
Sum of MUFA	29·54	26·30	28·69	27·68	28·54	2·26	0·87
16:2 *n*-6	0·28	0·36	0·38	0·36	0·38	0·05	0·61
18:2 *n*-6	4·27	4·33	5·10	5·34	5·80	0·38	0·07
18:3 *n*-6	0·16	0·15	0·16	0·15	0·16	0·01	0·97
20:3 *n*-6	0·16	0·16	0·20	0·17	0·18	0·01	0·62
Sum of *n*-6 PUFA	5·29	5·34	6·15	6·40	6·90	0·48	0·15
18:3 *n*-3	0·96	1·07	1·35	1·49	1·65	0·09	< 0·01*
20:3 *n*-3	0·32	0·29	0·32	0·33	0·34	0·03	0·83
20:5 *n*-3	3·22	3·02	3·23	3·24	3·32	0·27	0·95
22:5 *n*-3	1·55	1·50	1·67	1·72	1·77	0·12	0·54
22:6 *n*-3	9·18	8·48	9·20	9·07	9·21	0·67	0·93
Sum of *n*-3 PUFA	15·57	14·79	16·18	16·26	16·74	1·21	0·81
16:3 *n*-4	0·27	0·25	0·27	0·26	0·27	0·02	0·93
Sum of EPA + DHA	12·40	11·51	12·43	12·31	12·53	0·94	0·94

VLC-PUFA, very-long-chain PUFA.

* Significant difference (*P* < 0·05).

† VLC-PUFA composition of TAG fraction is not analysed.

The VLC-PUFA composition of the skin PL fraction from salmon fed different dietary levels of VLC-Conc1 is presented in Fig. 1 and Table 8. The fatty acid composition demonstrated a clear linear increase in the *n*-3 VLC-PUFA composition as the levels of the *n*-3 VLC-PUFA concentrate included in the feed increased (Fig. 1 and Table 8). Among the different VLC-PUFA present in the concentrate, the content of 28:8 *n*-3 was highest (Table 1), which was also reflected in the skin (Fig. 1 and Table 8). The content of the VLC-PUFA 24:5 *n*-3 increased from a mean of about 45 µg/g tissue in the 0 % group to a mean of about 186 µg/g tissue in the 10 % group, while the content of 24:6 *n*-3 increased from approximately 125 µg/g tissue in the 0 % group to 201 µg/g tissue in the 10 % group. The content of 26:6 *n*-3 increased from approximately 5 µg/g tissue in the 0 % group to approximately 56 µg/g tissue in the 10 % group, whereas 26:7 *n*-3 and 28:8 *n*-3 increased from 1·4 and 14 µg/g tissue in the 0 % groups, respectively, to about 5·5 and almost 400 µg/g tissue in the 10 % group, respectively. Additionally, the VLC MUFA 24:1 *n*-9, which was also present in VLC-Conc1, increased from a mean of about 63 µg/g tissue in the 0 % group to about 80 µg/g tissue in the 10 % group (Table 8).

**Table 8.** VLC-FA composition of the PL fraction from salmon skin measured as µg/g tissue and presented as mean values and pooled standard error of the mean (sem) (*n* 3 tanks per diet group, with pooled samples of ten fish from each of the triplicate tanks per diet group, giving thirty fish in total from each group). Diet groups are named according to the percentage VLC-Conc1 included in the feed

	VLC-Conc1 (%)						
	0	2·5	5	7·5	10	Pooled sem	*P*
24:1 *n*-9	63·06^ab^	51·54^a^	70·32^ab^	72·39^ab^	80·15^b^	4·95	0·021*
24:5 *n*-3	44·64^a^	69·01^ab^	95·18^b^	139·70^c^	186·15^d^	6·11	< 0·001*
24:6 *n*-3	125·68^a^	131·47^a^	152·03^ab^	181·52^bc^	291·68^c^	6·68	0·001
26:6 *n*-3	5·07^a^	13·26^b^	25·80^c^	40·87^d^	54·67^e^	1·40	< 0·001*
26:7 *n*-3	1·43^a^	2·26^a^	3·02^ab^	3·40^ab^	5·61^b^	0·53	0·015*
28:8 *n*-3	14·18^a^	75·45^a^	162·85^b^	288·98^c^	395·50^d^	14·40	< 0·001*

PL, phospholipid; VLC-fatty acid, very-long-chain FA.

^a-e^Groups not sharing the same letter are significantly different.

* Significant difference (*P* < 0·05).

#### Skin morphology

**Fig. 2.**
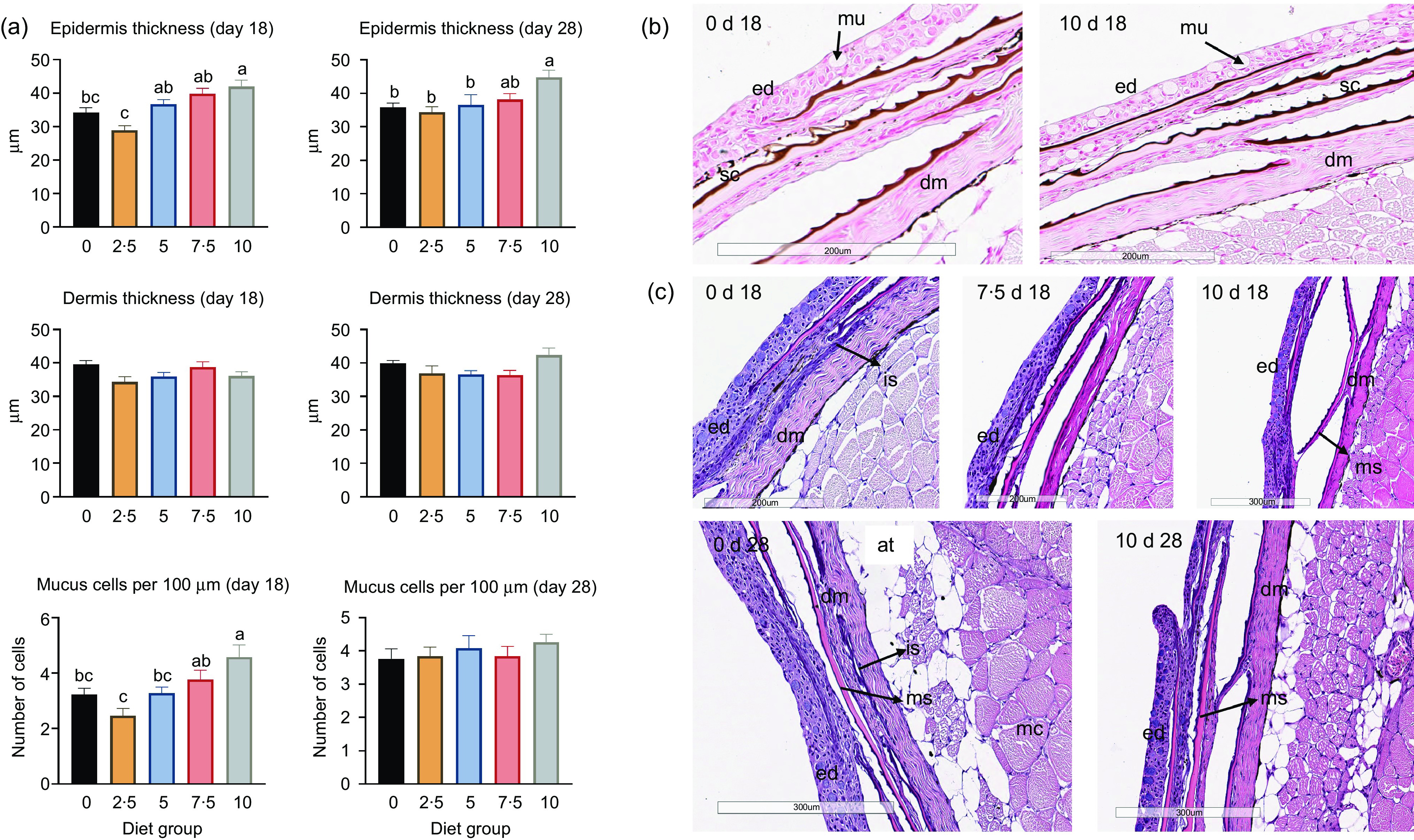
Histology of salmon skin from fish after feeding different dietary levels of VLC-Conc1. (a) Histological analysis of epidermis thickness, dermis thickness and mucus cell count per 100 µm (*n* 15 per group). Bars with different letters are significantly different (*P* < 0·05). (b) Histological images of skin samples stained with Von Kossa. Left image shows diet group 0 d 18, with light colouring of scales, right image shows diet group 10 d 18 with darker colouring of scales. (c) Histological images of skin samples stained with H&E. 0 d 18, diet group 0 d 18; 10 d 18, diet group 10 d 18; 7·5 d 18, diet group 7·5 d 18; 0 d 28, diet group 0 d 28; and 10 d 28, diet group 10 d 28. Small letters denote identified structures in the skin as follows: ed, epidermis; sc, scale; mu, mucus cell; dm, dermis; at, adipose tissue; is, immature scale; ms, mature scale; mc, muscle. (Von Kossa and H&E staining, 200× and 300× magnifications).

Increasing levels of the *n*-3 VLC-PUFA concentrate VLC-Conc1 in the feed led to a gradual increase in epidermis thickness at day 18, from 28·96 ± 1·30 µm in the 2·5 % group to 42·08 ± 1·84 µm in the 10 % group (*P* < 0·0001), and at day 28, from 34·30 ± 1·59 µm in the 2·5 % group to 44·79 ± 2·09 µm in the 10 % group (*P* = 0·0073) (Fig. 2(a)). Similarly, at day 18, there was a trend towards an increasing dermis thickness from the 2·5 % group to the 7·5 % group. At day 28, there were no significant differences in the thickness of the dermis between the groups. All groups showed increased thickness of the dermis at this point compared with that observed day 18. Furthermore, more mucus cells were detected in the higher percentage groups at day 18, with a significant difference between the groups (*P* = 0·0002). At day 18, the 2·5 % group had an average of 2·4 mucus cells per 100 µm, while the 10 % group had 4·6 per 100 µm. At day 28, there were no significant differences in the number of mucus cells between the diet groups. There were no morphological changes in mucus cell distribution, indicating that the fish had satisfactory environmental conditions and were not stressed.

Von Kossa staining revealed a darker colour of the scales in the 10 % diet group compared with that seen in the 0 % diet group (Fig. 2(b)). This method is used to visualise mineralisation; therefore, this finding demonstrates a more mature scale morphology in the 10 % diet group as it showed greater mineralisation. Furthermore, H&E staining showed thinner scales and more immature scales in the 0 % diet group at day 18 compared with the 7·5 % and 10 % groups, which demonstrated thicker and more mature scales (Fig. 2(c)). Differences between the groups were most obvious between the 0 % and 10 % groups, and histological differences were difficult to observe in the 7·5 % group. However, at day 18, immature scales were still present in the 0 % group, with some in the 7·5 % group but very few in the 10 % group. In general, fish in the 10 % group had a more mature scale structure at day 28 compared with those observed in the other groups.

#### *In vitro* salmon keratocyte migration in response to 26:6 *n*-3

**Fig. 3.**
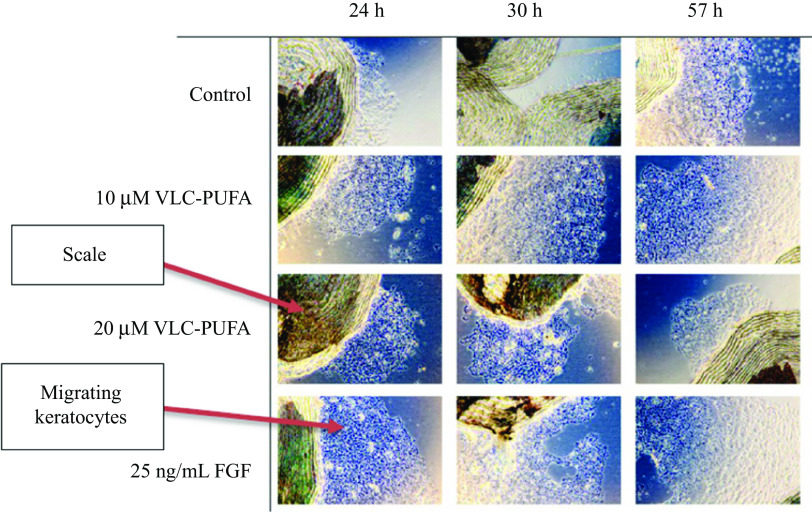
Cell migration from salmon scales. Images (phase contrast microscopy, 4× magnification) show a representative selection of scales from wells from the different groups taken at 24 h, 30 h and 57 h after plucking. Images are shown for the four experimental groups: control; 10 µM VLC-PUFA; 20 µM VLC-PUFA; and 25 ng/mL FGF. The VLC-PUFA 26:6 *n*-3 was used in the VLC-PUFA groups. VLC-PUFA, very-long-chain PUFA; FGF, fibroblast growth factor.

**Fig. 4.**
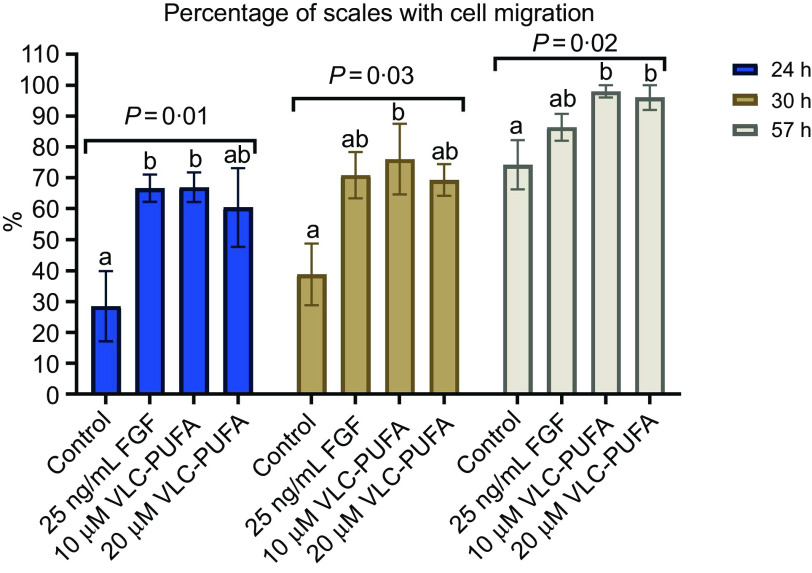
Effect of VLC-PUFA (26:6 *n*-3) on cell migration from salmon scales. Control (*n* 6), FGF (*n* 7), 10 µM VLC-PUFA (*n* 5) and 20 µM VLC-PUFA (*n* 5), where n represents the number of wells. Each well contained three to ten scales. The *y*-axis shows the percentage of scales with cell migration. Data are presented as the mean ± sem. Bars with different letters are significantly different (*P* < 0·05). VLC-PUFA, very-long-chain PUFA; FGF, fibroblast growth factor.

Salmon skin contains naturally low amounts of the *n*-3 VLC-PUFA 26:6 *n*-3 (Fig. 1). However, levels of this fatty acid increased significantly in the skin PL fraction in fish fed diets containing VLC-Conc1. Therefore, we examined the effect of this fatty acid on keratocyte migration. The results from the *in vitro* Atlantic salmon keratocyte migration experiment, in which scales were plucked from small fish to evaluate the migration of keratocytes from the scales, are presented in Fig. 3 and 4. We found a significant difference between the 10 μM and 20 μM VLC-PUFA (26:6 *n*-3) groups and FGF group compared with the control group (*P* = 0·01) after 24 h, where the control group had a lower migration of keratocytes from the scales. The same was seen 30 h after plucking (*P* = 0·03), and again, after 57 h, the VLC-PUFA groups showed a significantly higher percentage of scales with cell migration (*P* = 0·02).

#### Scratch assay and wound healing of human dermal fibroblasts supplemented with *n*-3 very-long-chain-PUFA

**Fig. 5.**
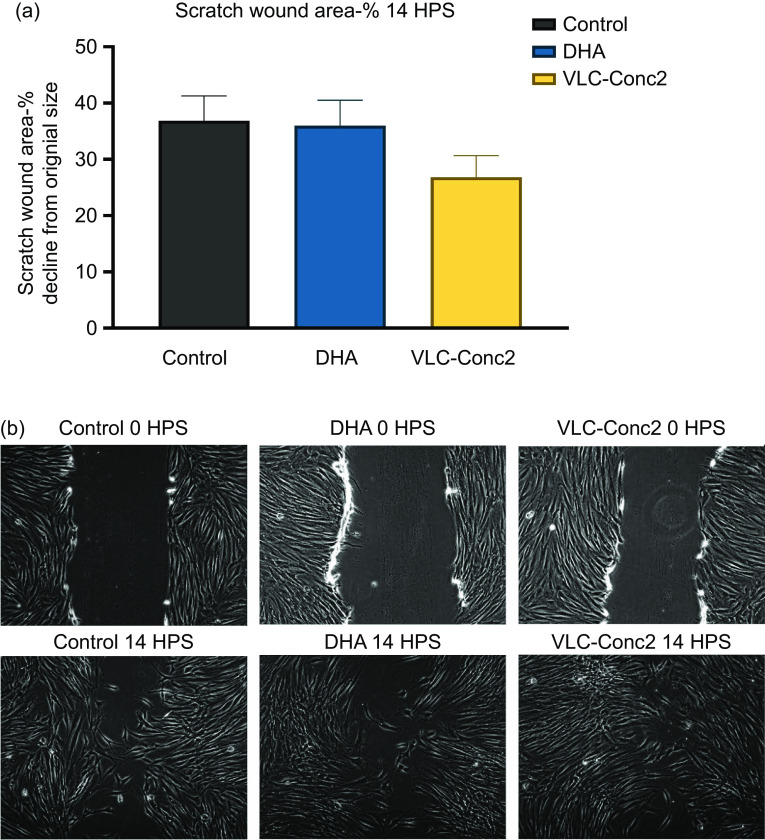
(a) Scratch assay analysis showing the percentage scratch size 14 h post-scratch. Data are calculated based on the respective initial scratch size of each well and presented as the mean of three experiments (*n* 3) and standard error of the mean (sem). Each mean is calculated from six wells per group in experiment 1 and 2, and in experiment 3, the control group consists of nine wells, and the 3 µM VLC-Conc2 and 3 µM DHA groups of six wells each. (b) Cell migration from 0–14 HPS. Images are from one well of each substrate group, illustrating the process. HPS, hours post-scratching.

The results from the scratch assay showed that the scratch size of the VLC-Conc2 group was approximately 10 % smaller at 14 HPS than those seen in the control and DHA groups, albeit not significant (*P* = 0·2597) (Fig. 5). This finding demonstrates a trend of faster migration of cells to the scratch area in the VLC-Conc2 group compared with these control groups.

**Fig. 6.**
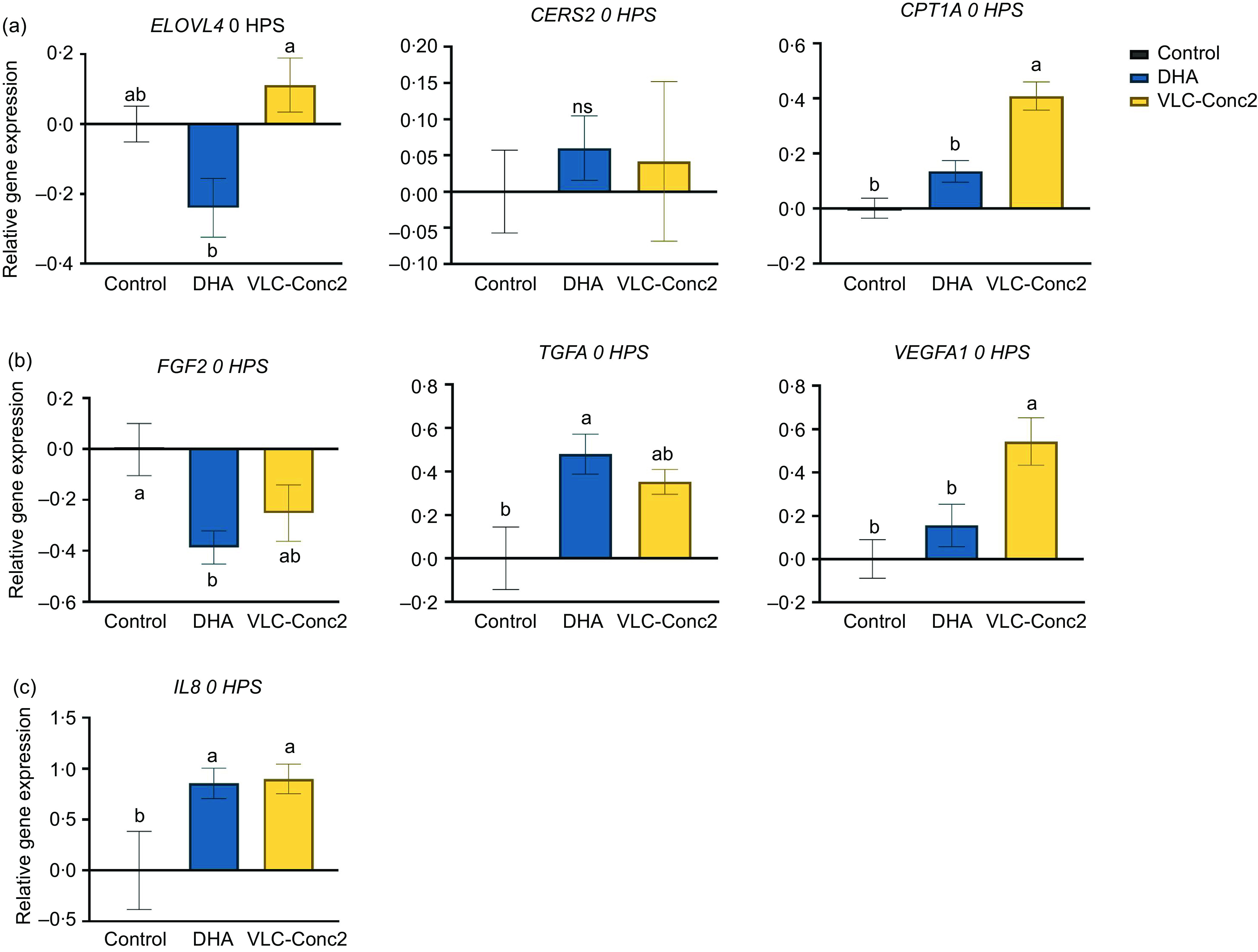
Gene expression in human dermal fibroblasts harvested immediately after scratch initiation (0 HPS). Data are presented as the mean ± sem of each group (*n* 6). Bars with different letters are significantly different (*P* < 0·05). HPS, hours post-scratching; NS, non-significant differences; ELOVL4, elongase of very long-chain protein-4; CERS2, ceramide synthase 2; CPT1A, carnitine palmitoyltransferase 1A; FGF2, fibroblast growth factor 2; TGFA, transforming growth factor A1; VEGFA1, vascular endothelial growth factor A1.

Cells in each group were incubated with the relevant fatty acids for approximately 24 h prior to creating a scratch. Gene expression analysis of human dermal fibroblasts harvested directly after creating the scratch (0 HPS) showed that expression of *ELOVL4*, which is involved in lipid metabolism, was significantly downregulated in the DHA group compared with the VLC-Conc2 and control groups (*P* = 0·0110), whereas carnitine palmitoyltransferase 1A (*CPT1A*), which is involved in mitochondrial β-oxidation of fatty acids, showed significantly higher expression in the VLC-Conc2 group compared with the other groups (*P* < 0·0001). There were no significant differences between the groups in the expression of ceramide synthase 2 (*CERS2*) at this time point (Fig. 6(a)). Genes involved in cell proliferation and cell growth were also affected. Expression of transforming growth factor *α* (*TGFA*) was significantly different between the groups, with the highest expression seen in the DHA group (*P* = 0·0145), and vascular endothelial growth factor A1 (*VEGFA1*) showed significantly higher expression in the VLC-Conc2 group compared with the other groups (*P* = 0·0044). Additionally, *FGF2* showed significantly lower expression in both the DHA and VLC-Conc2 groups compared with the control group (*P* = 0·0346) (Fig. 6(b)). Genes involved in the regulation of inflammation were also affected. *IL8* showed significantly higher expression in both the DHA and VLC-Conc2 groups compared with the control group (*P* = 0·0392) (Fig. 6(c)).

**Fig. 7.**
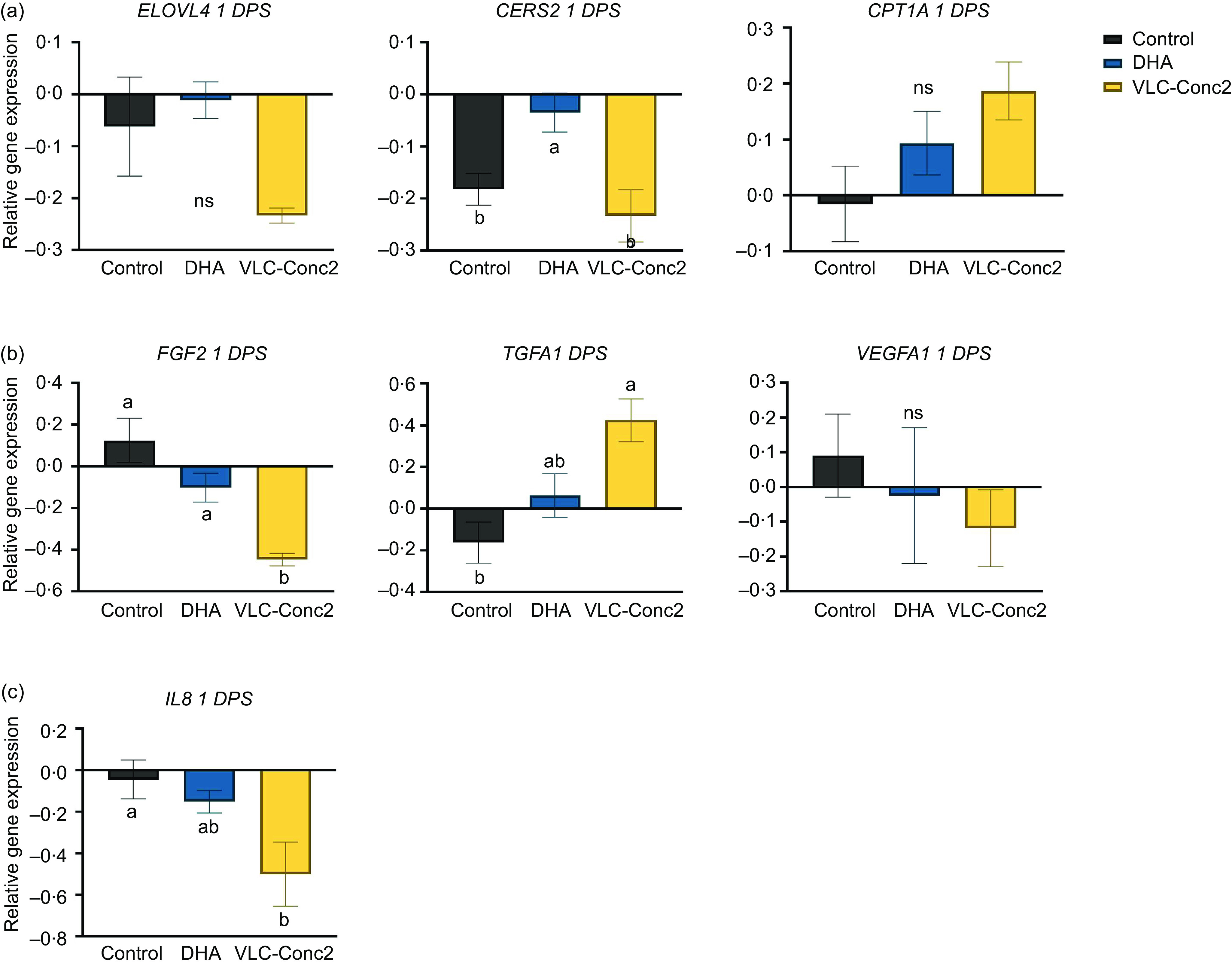
Gene expression in human dermal fibroblasts harvested at 1 DPS. Data are presented as mean ± sem of each group, where the DHA and VLC-Conc2 groups represent six individual wells in each group and the control group represents nine individual wells. Bars with different letters are significantly different (*P* < 0·05). DPS, days post-scratching; NS, non-significant differences; ELOVL4, elongase of very long-chain protein-4; CERS2, ceramide synthase 2; CPT1A, carnitine palmitoyltransferase 1A; FGF2, fibroblast growth factor 2; TGFA, transforming growth factor A1; VEGFA1, vascular endothelial growth factor A1.

Quantitative PCR analysis of the human dermal fibroblasts harvested at 1 DPS showed no significant difference in *ELOVL4*, *CPT1A* or *VEGFA1* expression. However, expression of *CERS2*, which is involved in lipid metabolism, was significantly lower in the control and VLC-Conc2 groups compared with the DHA group (*P* = 0·0086) (Fig. 7(a)). Genes involved in cell proliferation and cell growth were also affected at this time point, with significant down-regulation of *FGF2* in the VLC-Conc2 group compared with the DHA and control groups (*P* = 0·0008) and significant up-regulation of *TGFA1* in the VLC-Conc2 group compared with the control and DHA groups (*P* = 0·0028) (Fig. 7(b)). Furthermore, *IL8* was significantly downregulated in the VLC-Conc2 group compared with the other groups at this time point (*P* = 0·0206) (Fig. 7(c)).

**Fig. 8.**
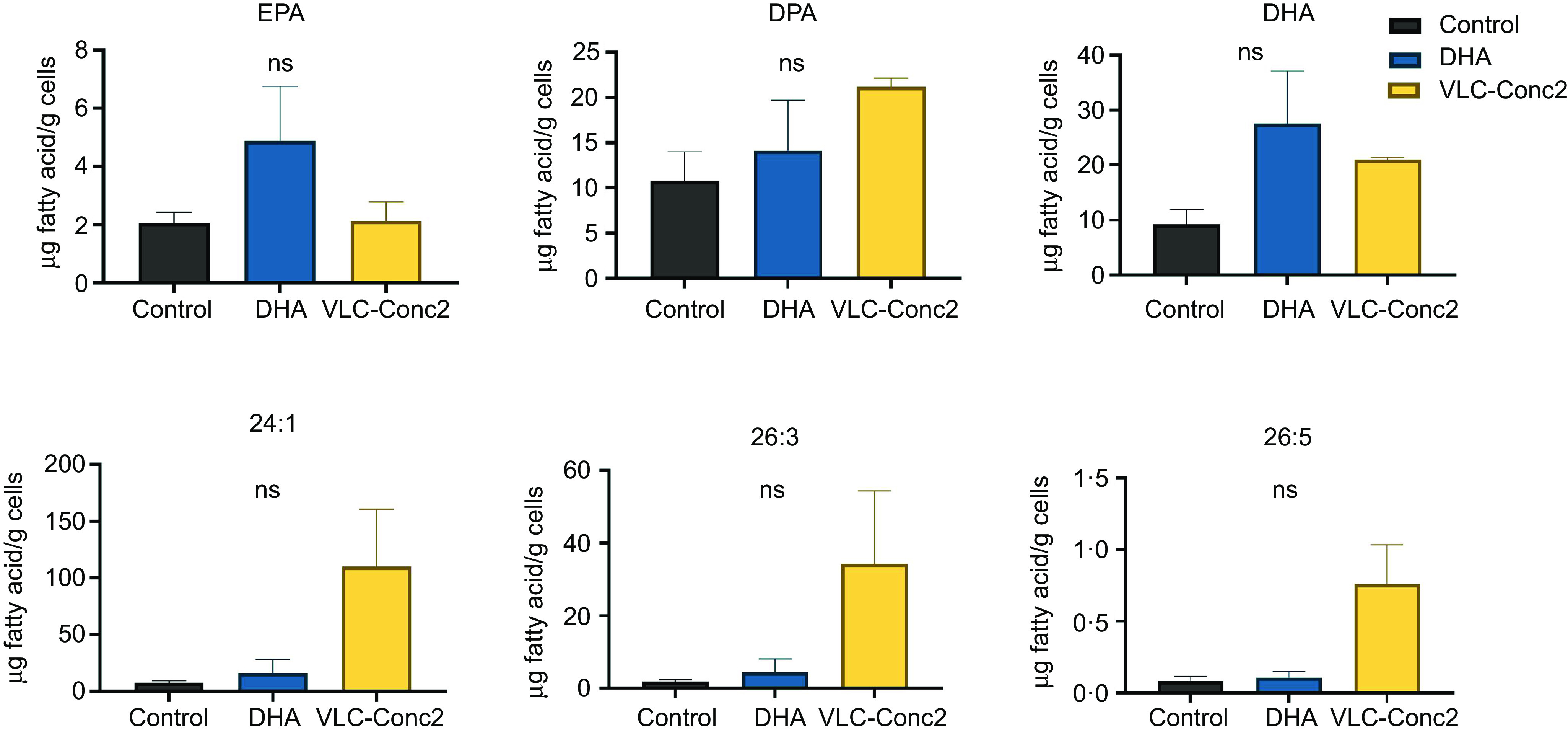
Long-chain fatty acid and VLC-PUFA composition of human dermal fibroblasts supplemented with either VLC-Conc2, DHA or albumin (control). NS, non-significant difference. VLC-PUFA, very-long-chain PUFA.

There were no significant differences in the fatty acid composition in terms of long-chain fatty acids and VLC-PUFA between the treatment groups (Fig. 8). However, there was a trend towards higher levels of the VLC-PUFA 26:3 and 26:5 *n*-3 in the VLC-Conc2 group. The MUFA 24:1, which was present in VLC-Conc2, also showed a trend to be higher in the VLC-Conc2 group.

## Discussion

The physiological effects of VLC-PUFA at the cellular level are not yet understood, although there is increasing evidence for their role in crucial body functions. VLC-FA are only present in the body after *in situ* synthesis from shorter chain precursors via the actions of ELOVL4^(38)^ and have previously not been available as food supplements or for research trials. We recently created a VLC-PUFA concentrate and showed that VLC-PUFA are bioavailable from the diet and deposited in various organs of mice and Atlantic salmon^(29)^. Previous studies have shown that mutations in the *ELOVL4* gene, which encodes an enzyme essential for the elongation of shorter-chain fatty acids to VLC-FA, lead to several tissue-specific disorders^(7,24,28,39,40)^. This raised the question whether it is possible to correct for deficient *in situ* production of VLC-FA via oral supplementation. Dietary supplementation studies also enable the study of biological functions. We reported that oral intake of VLC-PUFA led to the highest levels being deposited in skin tissue compared with eye, brain, liver and heart^(29)^. The aim of this study was to evaluate how dietary supplementation of VLC-PUFA affects skin functions, particularly in relation to skin maturation and wound closure.

We found that the VLC-FA present in the highest quantities in VLC-Conc1 were the same fatty acids that were deposited in the PL fraction of skin tissue of Atlantic salmon after oral intake of VLC-Conc1 for 4 weeks, thus greatly reflecting the dietary fatty acid composition. The VLC-PUFA concentrate (VLC-Conc1) had highest levels of the VLC-PUFA 28:8 *n*-3, which was also the VLC-PUFA that was increased the most in the skin. This fatty acid is naturally present in very low levels in the skin. Similarly, levels of 26:6 *n*-3 also increased significantly in the skin after oral intake of VLC-Conc1. This finding is consistent with previous studies that demonstrated that the fatty acid composition of skin is strongly affected by the fatty acid composition of the diet^(41)^. However, the fatty acids 24:5 *n*-3 and 24:6 *n*-3 were also present to a small degree in the skin tissue of the diet group not given any VLC-Conc1 in their feed (0 % group), which could be an indication of endogenous production. Another possibility is that these fatty acids are both intermediates in the synthesis of EPA to DHA, which could also explain why they were detected in the skin of this diet group. Nonetheless, the content of both these fatty acids increased linearly after oral intake of VLC-Conc1, even though the EPA and DHA levels were balanced between the different dietary groups, and there were no significant differences in EPA or DHA content in the skin tissue between the different diet groups This indicates that the majority of the 24:5 and 24:6 fatty acids in the skin most likely originates from the diet.

The thickness of epidermis of the skin was increased in the skin of juvenile Atlantic salmon from start to the end of the feeding trial in all the diet groups. A general increase in epidermis thickness over time is likely attributable to the increased weight of the fish between these measurements. However, differences between the diets were also detected. At day 18, the epidermis in the 10 % VLC-Conc1 group was on average 7·9 µm thicker than that of the 0 % VLC-Conc1 group, whereas this was on average 8·9 µm thicker at day 28. There were also more mucus cells in the 10 % group, which is most likely due to the increased thickness of the epidermis. The 10 % group had on average 1·35 mucus cells per 100 µm more than the 0 % group. Additionally, the 10 % group had more mineralised scales compared with the lower percentage groups, which is an indication of a more rapid maturation of the scales. The observed positive effect on scale mineralisation is interesting in terms of skin health and skeletal mineralisation. EPA and DHA have previously been shown to influence mineralisation in human osteoblasts^(42)^ and to stimulate precursor cells from Atlantic salmon to differentiate to osteoblasts *in vitro* ^(43)^, and several studies promote fish scales as a unique and useful model in mammalian bone research, suggesting that the teleost scale is very similar to mammalian bone^(43,44)^. In our study, fish in all the diet groups were given relatively high amounts of EPA and DHA, yet they demonstrated enhanced mineralisation of scales with additional supplementation with VLC-Conc1. Thus, based on our findings, dietary supplementation with *n*-3 VLC-PUFA could enhance bone mineralisation. The observed increase in epidermis thickness, mucus cell count and more rapid scale development in the high VLC-PUFA groups indicate that skin of fish in the VLC-PUFA diet groups developed more rapidly, which may provide them with more robust skin earlier in development. Robinson *et al.* ^(45)^ reported the development of salmon skin and bone and emphasised the increase in epidermis thickness and number of mucus cells, as well as mineralisation, as important developmental factors and indicators of a healthy fish. The more rapid maturation of the scales adds to this, as it is part of the protective barrier of the fish^(9)^. These findings could be of importance in aquaculture, since the transfer of salmon from freshwater to seawater induces stress and challenge the skin integrity, making it especially vulnerable to wounds during this phase^(46,47)^.

We further examined the effect of VLC-PUFA on the migration of Atlantic salmon keratocytes using an *in vitro* model to assess the potential effects of VLC-PUFA on wound healing. Keratinocytes and keratocytes are human and fish skin cells, respectively, and are activated early post-injury to migrate across the wound to reform the epidermal layer in a process called re-epithelialisation^(3,48)^. In fish, motile keratocytes play an important role in the wound healing process by migrating across the wounded areas to provide a protective barrier^(3)^. In this study, we demonstrated that primary keratocytes migrated more rapidly from scales from freshwater Atlantic salmon in cell culture when supplemented with the *n*-3 VLC-PUFA 26:6 compared with the controls. We selected 26:6 as this was one of the VLC-PUFA that increased the most in the skin tissue after feeding fish the VLC-Conc1 and since this VLC-PUFA is not normally detected in the skin. These findings indicate that *n*-3 VLC-PUFA may have a stimulatory effect on the cell migration processes in fish and may therefore have a positive effect on wound closure. From our findings, supplementation with VLC-PUFA may improve skin tissue functions in Atlantic salmon, especially in the early phases of development. This finding is in addition to the existing levels of EPA and DHA present in the skin of the fish in all dietary groups, both of which are known to have important functions for the robustness of Atlantic salmon^(49)^. However, further studies are required to establish how this translates to human skin and bone health.

Human wound healing can be divided into three main stages: inflammation, migration/proliferation and maturation^(50)^. A characteristic feature of chronic wounds is the failure to re-epithelialise, which is most often caused by hampered migration capacity of keratinocyte/keratocytes and/or fibroblast migration. Migration failure may occur due to a lack of functional extracellular matrix, excessive inflammation and protease activity, altered expression and distribution of cytokines^(51)^, exposure to chemical components^(32)^, or stress^(52)^. Previous studies have shown that skin wound healing processes in humans depend on the migration of keratinocytes and fibroblasts to the wound bed to close the wound^(14)^, indicating that *in vitro* cellular migration models may be used as a model for wound healing. We examined the effect of VLC-PUFA on the migration of human dermal fibroblasts to a scratch wound to understand how VLC-PUFA can influence wound healing in humans. DHA has many active metabolites, and several bioactive metabolites are also involved in wound healing and tissue regeneration^(53)^; thus, DHA was used as a positive control. We observed more rapid migration of fibroblasts to the scratched area after supplementation with VLC-Conc2 compared with the control and DHA-supplemented control group, indicating that VLC-PUFA have greater effects than DHA on the rate of fibroblast migration to the wound site, which is in agreement with the finding that VLC-PUFA increased migration of salmon keratocytes.

The scratch assay principally covers the second stage of wound healing, which is characterised by the proliferation and migration of keratocytes and fibroblasts^(54)^. When wounded or scratched, cell monolayers respond to disruption of cell–cell contact by initiating the proliferation and migration of different cell types, such as fibroblasts and keratinocytes, through alterations in growth factors and cytokine expression^(55–57)^. We observed a significant increase in the gene expression of *IL8* initially after creating the scratch in both the DHA and VLC-Conc2 groups compared with the control group, while expression was significantly downregulated after 24 h in these groups. IL8 is a chemoattractant cytokine that plays an important role in the early stage of wound healing by acting as an attractant for cell migration to the wound site, starting with the proliferative phase/recruitment and replication of cells, which are necessary for tissue regeneration^(57)^. The subsequent down-regulation of IL8 is logical as the scratch starts to close and the need for cell migration declines. Furthermore, VEGFA and FGF2 are positive regulators of angiogenesis, which occurs during the second stage of wound healing. Our data showed that expression of *VEGFA1* was significantly higher in the VLC-Conc2 group compared with the DHA and control groups initially after creating the scratch (0 HPS). Furthermore, expression of *FGF2* was significantly different between the control and DHA groups, with the lowest expression in the DHA group at 0 HPS. At 1 DPS, there was a significant difference in the expression of *FGF2* in the VLC-Conc2 group compared with control and DHA groups, with the lowest expression observed in the VLC-Conc2 group. This is a logical observation, as FGF2 is known to induce cell proliferation^(58)^ and because the VLC-Conc2 group showed the greatest closure of the scratched area at this time point. LC-PUFA are known to alter the production of pro-inflammatory cytokines, which are involved in coordinating the molecular and cellular processes that occur during the inflammatory stage of wound healing, in which the key aim is to prevent infection^(59)^. The *n*-3 LC-PUFA EPA and DHA were previously shown to affect the production of pro-inflammatory cytokines, which act as local inflammatory mediators and can thus regulate the wound healing process^(60)^.

While several studies have shown that PUFA have beneficial effects in wound healing assays^(21)^, to our knowledge, our findings are the first to demonstrate the beneficial effects of VLC-PUFA in wound healing and cell migration. The present study contributes to the scarce literature on VLC-PUFA and highlights their potential effects on skin and skin health, in both human and animal models. It is reasonable to assume that these unique fatty acids, although low in abundance, have some essential effects in the different organs and tissues in which they normally appear, considering the pathology observed in relation to *ELOVL4* mutations. Based on the *in vivo* and *in vitro* observations presented in the present study, with similar findings in both human and fish, it is possible that VLC-PUFA have positive effects on skin tissue development, function and integrity. However, future studies are needed to further assess the clinical relevance of VLC-PUFA in skin tissue and determine whether dietary supplementation of VLC-PUFA may contribute to more robust skin and faster wound healing *in vivo*.

## Conclusions

The findings of this study demonstrate the uptake of VLC-PUFA from the diet into the skin tissue of Atlantic salmon, with a positive effect on skin development and maturation in terms of increased epidermis thickness, mucus cells, and scale development and mineralisation *in vivo*. Furthermore, supplementing human and fish skin cells with VLC-Conc2 and the VLC-PUFA 26:6 *n*-3, respectively, resulted in increased cell migration *in vitro*. Taken together, these results suggest that VLC-PUFA may have beneficial effects on skin tissue development, function and integrity, which could be important for skin health. These preliminary findings provide the basis for further verification studies.
