# Investigation of the functions of *n*-3 very-long-chain PUFAs skin using *in vivo* Atlantic salmon and *in vitro* human and fish skin models – ADDENDUM

**DOI:** 10.1017/S0007114524000151

**Published:** 2024-05-14

**Authors:** Martina Torrissen, Elisabeth Ytteborg, Harald Svensen, Iren Stoknes, Astrid Nilsson, Tone-Kari Østbye, Gerd Marit Berge, Marta Bou, Bente Ruyter

Martina Torrissen’s email address has changed since the initial publication of this article. Please could all correspondence be sent to martina.torrissen@gmail.com.

## References

[ref1] Torrissen M , Ytteborg E , Svensen H , et al. Investigation of the functions of n-3 very-long-chain PUFAs in skin using in vivo Atlantic salmon and in vitro human and fish skin models. British Journal of Nutrition. 2023;130(11):1915–1931. 10.1017/S0007114523001150 PMC10630148 37169355

